# Integrating Self-Management Education and Support in Routine Care of People With Type 2 Diabetes Mellitus: A Conceptional Model Based on Critical Interpretive Synthesis and A Consensus-Building Participatory Consultation

**DOI:** 10.3389/fcdhc.2022.845547

**Published:** 2022-06-03

**Authors:** Claudia Huber, Chantal Montreuil, Derek Christie, Angus Forbes

**Affiliations:** ^1^ HES-SO University of Applied Sciences and Arts Western Switzerland, School of Health Science Fribourg, Fribourg, Switzerland; ^2^ Florence Nightingale Faculty of Nursing, Midwifery & Palliative Care, King’s College London, James Clerk Maxwell Building, London, United Kingdom; ^3^ Institute of Higher Education and Research in Healthcare, University of Lausanne, Lausanne, Switzerland

**Keywords:** integration, self-management education and support, critical interpretive synthesis, theory development, complex adaptive systems, type 2 diabetes

## Abstract

The integration of self-management education and support into the routine diabetes care is essential in preventing complications. Currently, however, there is no consensus on how to conceptualise integration in relation to self-management education and support. Therefore, this synthesis presents a framework conceptualising integration and self-management.

**Methods:**

Seven electronic databases (Medline, HMIC, PsycINFO, CINAHL, ERIC, Scopus and Web of Science) were searched. Twenty-one articles met the inclusion criteria. Data were synthesised using principles of critical interpretive synthesis to build the conceptual framework. The framework was presented to 49 diabetes specialist nurses working at different levels of care during a multilingual workshop.

**Results:**

A conceptual framework is proposed in which integration is influenced by five interacting components: the *programme ethos* of the diabetes self-management education and support intervention (content and delivery), *care system organisation* (the framework in which such interventions are delivered), *adapting to context* (the aspects of the people receiving and delivering the interventions), *interpersonal relationship* (the interactions between the deliverer and receiver of the intervention), and *shared learning* (what deliverer and receiver gain from the interactions). The critical inputs from the workshop participants related to the different priorities given to the components according to their sociolinguistic and educational experiences, Overall, they agreed with the conceptualisation of the components and their content specific to diabetes self-management education and support.

**Discussion:**

Integration was conceptualised in terms of the relational, ethical, learning, contextual adapting, and systemic organisational aspects of the intervention. It remains uncertain which prioritised interactions of components and to what extent these may moderate the integration of self-management education and support into routine care; in turn, the level of integration observed in each of the components may moderate the impact of these interventions, which may also apply to the impact of the professional training.

**Conclusion:**

This synthesis provides a theoretical framework that conceptualises integration in the context of diabetes self-management education and support in routine care. More research is required to evaluate how the components identified in the framework can be addressed in clinical practice to assess whether improvements in self-management education and support can be effectively realised in this population.

## Introduction

A large number of people living with type 2 diabetes mellitus – a debilitating long-term metabolic condition – develop chronic complications related to micro- and macrovascular damage (1). These severe long-term health deficits are hallmarks of the length and gravity of metabolic dysfunctions in type 2 diabetes (2). In 2015, the Global Burden of Disease Study identified diabetes as the 6^th^ leading cause of disability, with an increasing incidence among people of working age (3). This global rise of diabetes disability is mostly due to type 2 diabetes, which accounts for 90% of the estimated 422 million people living with diabetes worldwide (4). This means that more and more people are living more years with functional health impairments – which has far-reaching implications for the wellbeing of individuals and their families, and also for the delivery of health care services and the financing of health systems.

It is well known that lowering blood glucose levels has beneficial effects on micro- and macrovascular complications and that people benefit from early detection and the intensive patient-centred management of type 2 diabetes (5–8). However, as a recent study conducted in a large integrated health care system showed, in more than half of their study population the initiation of antihyperglycemic therapy was delayed by at least 6 months; and this despite evidence that delayed therapy initiation is generally associated with poorer glycaemic control and a higher risk of complications (9).

Besides prescription medicines, there is strong evidence that diabetes self-management education and support (DSMES) is effective in improving glycaemic control and patient-related outcome measures, especially when combined with psychosocial interventions, as well as being cost-effective (10–13). However, despite the demonstrated benefits, participation in DSMES remains low (14–16). This strongly suggests that DSMES is not yet fully integrated into routine health care although it is recommended in most national and international guidelines (17). Higher attendance rates in DSMES were observed in systems with structured health care provision. For example, disease management programmes with structured DSME for type 1 and type 2 diabetes were introduced in Germany in 2003. There are similarities between DSMES in type 1 and in type 2 diabetes, however approaches used vary considerably between the different types of diabetes and the target populations (18). The focus of type-2-diabetes DSMES is often on lifestyle changes (exercise and weight loss), which is distinct from the focus in type 1 diabetes. As the 2020 evaluation of the type 2 diabetes cohort in Germany showed, DSMES participation rates in the age group up to 30 years increased to almost 60% in the first year of enrolment in a disease management programme, with a high drop over the following years (19). This indicates that more integrated structures and continuous support may be important features to improve the uptake and outcome of DSMES in both type 1 and type 2 diabetes. The DSMES interventions have multiple facets influenced by the characteristics, activities and interactions of the people living with diabetes, the healthcare professionals involved in delivery, and the health care systems themselves – all of which may hinder or facilitate the integration of a person-centred approach to DSMES into routine care (20). There is a wide agreement that such an approach respects and responds to needs, values and preferences of people with diabetes and that their values should guide the clinical decision process (21). However, there is less understanding on how to implement this approach in routine care. For example, whilst person-centredness is a core element of care, in daily practice healthcare professionals may be ambivalent about encouraging people with diabetes to express their emotional and psychosocial concerns in the limited time they have available during consultations (22, 23). This relative disregard highlights the importance of creating conditions that include person-centredness in care delivery. Such conditions are generally advocated in integrated care systems which are considered essential in improving care for people with chronic conditions who require ongoing care and support (24–26).

Integrated care is largely expected to achieve better outcomes, experiences and use of resources through shared responsibilities of healthcare professionals coordinated across care facilities and support systems (27, 28). In many health care systems, evaluations of integrated care interventions have shown equivocal results due to the broad range of activities and concepts associated with integrated care, which is a complex concept with no agreed definition (29, 30). The concept has been related to integrated care processes that build a whole-system approach of coordinated care delivery, as well as conceptual components that share an integrated whole-care philosophy of healthcare professionals’ collaboration (28, 29, 31–33). For the purpose of this study, this widely used definition of integration in health care delivery is used: “a coherent set of methods and models on the funding, administrative, organisational, service delivery and clinical levels designed to create connectivity, alignment and collaboration within and between the cure and care sectors” (32). These multiple interactions may be affected by the different underlying approaches of healthcare professionals providing elements of DSMES within a fragmented delivery of health services that may further impede person-centred support (34–37). To address these shortcomings, it is important to understand these multiple interactions and identify the essential components of integration in the context of DSMES in routine care.

This study uses the theory of complex adaptive systems (CAS) to explore the different components of integrating DSMES in the often complex and multifaceted situations in routine care. The CAS’s main features are emergent behaviours, self-organisation, co-evolution using simple rules and non-linear processes that represent interacting systems in health care (38–40). These perspectives are used in contexts that face disordered and uncertain conditions to understand the behaviour of systems and identify components that explain their interactions and relationships (41–49). Several aspects of the CAS perspective have been used, for example, to explore the transformative processes in the experiential participatory learning processes of DSMES and identity the mutual influences of healthcare professionals and people with diabetes towards a new context-specific relationship (50–53). These relationships continue to develop during interactions, shaping the kind of consensus-building required in the processes of shared decision-making and promoting the exchange of skills and competencies (54, 55). Considering these features helps understand the multiple dynamic interactions influencing DSMES and the level of integration along the different axes of integrated care which are also considered in the Chronic Care Model (32, 56).

This study aims are to conceptualise integration in relation to DSMES from a CAS perspective and to propose a theoretical model that can serve as starting point for developing a shared understanding of integration within the healthcare professional community.

## Methods

The guidance for Undertaking Reviews in Health Care was used to inform the development of the search strategy, identify inclusion and exclusion criteria, select studies and extract the data (57). Principles from critical interpretive synthesis were used for data synthesis (58). This transformation-based approach incorporates elements of conventional systematic reviews with an interpretative and critical approach to data synthesis and is well suited to developing new theoretical models (59). Within this configurative qualitative evidence synthesis, concepts from included qualitative and quantitative articles were extracted and examined across studies in terms of similarities and differences to develop a theoretical proposal (60). This approach of synthesising concepts allowed the researchers to build a line of argumentation, define the synthesising “integration” argument and develop a model of integrated self-management education and support in routine care.

### Review Question and Searching the Literature

Several preliminary searches identified that the term “integration” was mainly related to integrated care with tendencies towards linking similar levels of care (multi-professional teams) and different levels of care delivery (primary, secondary and tertiary care) to collaborate in and coordinate the processes of integrated care for improved continuity, access, quality, user satisfaction and efficiency. However, some studies reported conditions and features of DSMES to extrapolate data for developing the components of integration in this context. After team discussion, the review was guided by the following review question: What are the essential components of integration in relation to DSMES and how do they manifest themselves (considering structures of DSMES, context and participant experience)?

The search strategy was developed in collaboration with a librarian according to the principles of critical interpretive synthesis and used to specifically identify articles relevant to the conceptualisation in the following databases from 2004 to 2014 with a search update in January 2022: 1) Medical Literature Analysis and Retrieval System Online (Medline), Current Index to Nursing and Allied Health Literature (CINAHL), Psychological Information Database (PsycINFO), Health Management Information Consortium (HMIC), Education Resources Information Centre (ERIC), Scopus, Web of Science; 2) hand-searching of the bibliographies of retrieved articles and grey literature and 3) identifying articles through colleagues with experience in this field (58, 61). The databases were searched for relevant literature, using both the Medical Subject Headings (MeSH) and free-text keywords that referred to self-management, education and support, type 2 diabetes, integration, and interdisciplinary teams. Validated methodological filters for capturing quantitative and qualitative articles were identified by consulting the librarian and various guidelines (62, 63). The syntax of the search terms was adapted for each database. The citations were exported to Endnote (version x7), and all duplicates were removed.

### Inclusion and Exclusion Criteria

The inclusion and exclusion criteria were developed to capture empirical studies (qualitative and interventional study designs) representing components of integration in relation to DSMES into routine care. The main inclusion criteria were a description of the integration of DSMES into routine care and/or research evidence to extrapolate such an integration. The articles were limited to those that a) described at least two instances of integration (considering structures, context and participant experiences); b) included a clear description or definition of DSMES based on research evidence; c) defined linkages to an interprofessional team (including peers); d) demonstrated continuous DSMES in different settings of care delivery with evident linkages to a community, to primary/secondary/tertiary care or to a virtual network for ongoing support; and e) addressed DSMES to adult participants (≥ 18 years of age) with diagnosed type 2 diabetes and defined the healthcare professionals who delivered DSMES to these participants. Articles with access to the full text were included. Articles published before 2004 or in languages other than English, French and German, or without an English abstract, were excluded.

### Sampling

All titles and abstracts were initially assessed by one reviewer for their relevance to contributing to the conceptualisation of the integration of DSMES into routine care. At this stage, articles were rated as “irrelevant”, “of uncertain relevance” or “probably relevant” using the inclusion/exclusion criteria. A random selection of 5% was checked for relevance by a second reviewer. The articles rated as “of uncertain relevance” and “probably relevant” were further assessed. A formal test of interrater agreement was not conducted, but the results from each reviewer were discussed in two meetings and all articles were compared in detail in terms of their theoretical contribution to the conceptualisation until a high level of mutual agreement had been reached. According to the principles of a critical interpretive synthesis, articles were included based on their conceptual quality, which means that the identified articles provided content relevant to the review questions (58).

### Determination of Quality

The articles were assessed using the five quality criteria associated with the likely relevance of an article as described for critical interpretative synthesis (58): 1) clearly stated aim and objectives of the research; 2) clearly specified research design, appropriate for the aims and objectives of the research; 3) researchers provided a clear account of the process by which their findings were produced; 4) enough data displayed to support their interpretations and conclusions; and 5) appropriate method of analysis and adequate explication (64). Consistent with this approach, articles with lower relevance or methodological limitations were discussed by the reviewers.

### Data Extraction

The following data were extracted from the selected articles: author, year, country, study design, purpose/aim, sample setting, participants (*i.e.* receivers of the intervention) and healthcare professionals (*i.e.* deliverers of the intervention); in addition, the following information was extracted from the quantitative articles: intervention, including theoretical framework, follow-up, control, primary outcome and, if available, patient-reported outcomes; and from the qualitative studies: participant eligibility criteria, recruitment context, data collection methods, data analysis, and identified themes and sub-themes.

### Data Analysis and Synthesis

The data from the full-text articles were coded for pertinent information by one researcher and 5% of the data coding was reviewed by a second researcher. After the coding of each article, a critique reflecting the comments of each article was recorded to capture the different ways in which the literature had conceptualised the integration of DSMES into routine care. To facilitate the processes of discovering the themes and patterns emerging from the articles, the data were imported into NVivo 11 software for sorting, classifying, and arranging the information. The synthesis in this review was organised around the core construct of integration related to DSMES in routine care.

The synthesis used four overlapping steps as transforming techniques for qualitative and quantitative articles (58, 65). First, the coding was analysed to identify themes that capture the content of integration from the relevant information of the articles. Second, the themes were compared with the data of each article and the initial concepts examined in terms of similarities and differences across articles. Third, the patterns were expressed as a transformed conceptualisation, that in critical interpretive synthesis is known as a synthetic construct. This developed as a critique of the literature, expressing contradictions and flaws in the evidence. Fourth, the identified concepts and critique were integrated into a theoretical framework in order to produce the synthesising argument which links the constructs to the themes. The synthesis integrates the evidence with the components of integration and interprets their interactions in the context of DSMES in routine care. The synthesis was done by one researcher and regularly discussed with a second researcher and team members to interpret the emerging findings. The saturation of themes and their repetition across data sets were discussed among researchers who used reflexivity at each stage of the data analysis and synthesis (66).

### Establishing Face Validity of the Theoretical Model

With the conceptual model having been developed through the literature and input from experts, the model was then presented and discussed in the nurse community to assess its face validity and acceptability (67). The conceptual model was presented and discussed in a workshop with diabetes specialist nurses working in primary, secondary and tertiary care in Switzerland. The workshop was simultaneously translated between German and French. The workshop participants were asked for verbal informed consent before the workshop started, explaining to them that their participation entailed the discussion of the conceptual model by answering two open-ended questions. The rounds of discussion were analysed directly during the workshop using a concept mapping approach (68. With this approach, concept mapping and note-taking were shared with the participants for immediate feedback at the end of the workshop.

Before the workshop, the model of integrated self-management education and support was briefly explained to the participants (10 minutes), then the workshop was opened by asking two open-ended questions:

1. How and why (or why not) do the components represent integration in relation to DSMES from your viewpoint?
2. How would you rate the importance of each of the components and which additional components, that promote self-management, are missing in the model from your viewpoint?

The participants first discussed each question in small groups of a maximum of 5 people (sitting at the same table) and then one person from each group summarised the most important points and presented them to the whole group while two facilitators wrote down the keywords (2 x 15 minutes). Afterwards, the components were discussed in the whole group, synthesised and validated with regard to the linguistic subtleties and the different interpretations for an additional 15 minutes. The facilitators gave the participants feedback of their understanding with simultaneous translation between German and French. The participants added commentaries until consensus was reached within and between the two linguistic groups.

## Results

The electronic bibliographic database search yielded 3709 articles for screening after excluding 132 duplicates and adding four records identified through other sources. Full texts were obtained for 227 articles, of which 206 articles were excluded for the following reasons: intervention delivered by one profession without a connecting network, one-time intervention, insufficient description.

**Figure 1:**
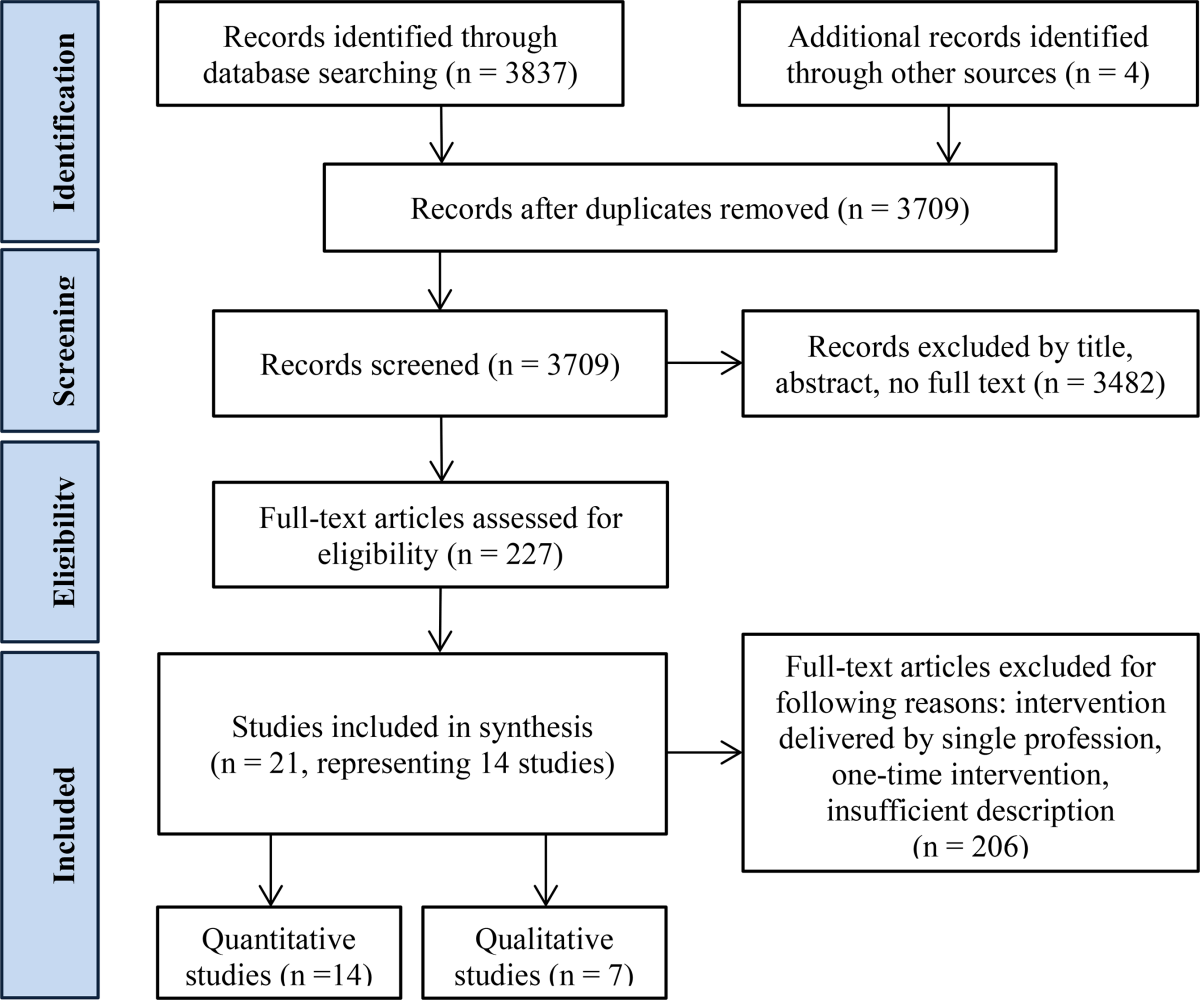
Study selection using modified PRISMA flow chart.

Altogether, 21 articles were found to be relevant for conceptualisation, of which 14 had interventional study designs, and seven were qualitative. These 21 articles represented 14 studies covering DSMES in the following settings: community care, primary care, secondary care, tertiary care, and pharmacies. The flow chart in **Figure 1** illustrates the study selection process (69).

### Characteristics of Relevant Studies

The articles all originated from industrialised countries: Australia (70, 71), Belgium (72, 73), France (74, 75), Italy (76), the Netherlands (77), Norway (78, 79), South Korea (80), Sweden (81–83), the UK (84–86) and the USA (87–90). The characteristics of the study designs, contexts and participants, together with the key concepts and themes related to integration, are presented in tables as **Supplementary Material**. The qualitative articles had clear research aims and an adequately described methodology. The quality score of the quantitative studies varied from medium to low. Because in critical interpretive synthesis conceptual relevance is more important than methodological rigour (58), all selected articles were included in the review. The contributions of each article to the conceptualisation of the components of integration are represented in **Table 1**.

**Table 1:** Contributions of the studies to the conceptualising of the components of the integration of DSMES into routine care.

Source	Interpersonal relationship	Programme ethos	Shared learning	Adapting to context	Care system organisation
Adolfsson et al. (82)	Peer support	Empowerment	Joint understanding	n/s	n/s
Adolfsson et al. (81)	Roles and positions	Empowerment	Joint understanding	Varieties of learning	n/s
Balcou-Debussche & Debussche, (74)	Disease responsibility	Problem-solving	Transfer of learning	Different locations	n/s
Carey et al. (84)	Roles and positions	Combined strategies	Joint training	n/s	n/s
Deakin et al. (85)	Peer support	Empowerment	Joint training	Community access	n/s
Debussche et al. (75)	n/s	Problem-solving	Collaboration in multi-professional team	Linguistic/cultural variations	Recall system
Du Pon et al. (77)	Person-centred communication	Empowerment	Joint training	Local access to care	Regular follow-up
Glasgow et al. (87)	n/s	Combined strategies	Individualised support strategy	IT resources	Service redesign
Glasgow et al. (88)	n/s	Combined strategies	Virtual and individualised support	Information on local options	Service redesign
Goderis et al. (73)	n/s	Combined strategies	Interdisciplinary training	Local need	Service redesign
Goderis et al. (72)	Person-centred communication	Combined strategies	Interdisciplinary training	Feedback on performance	Treatment guideline
Hepworth et al. (70)	Relationships in multi-professional care	Empowerment	Collaboration in multi-professional team	Local access to care	Regular follow-up
Katon et al. (89)	Person-centred communication	Problem-solving	Collaboration in multi-professional team	Coordination of care needs	Treatment guideline
Ko et al. (80)	Social support	Cognitive behavioural therapy	Collaboration in multi-professional team	Local need	Recall system
Mandalia et al. (86)	Peer support	Combined strategies	Joint understanding	n/s	n/s
Piatt et al. (90)	n/s	Empowerment	Joint training	Local need	Treatment guideline
Russell et al. (71)	Relationships in multi-professional care	Empowerment	Joint training	Local access to care	Service redesign
Rygg et al. (79)	Social support	Combined strategies	Joint understanding	Local access to care	n/s
Rygg et al. (78)	n/s	Combined strategies	Joint training	Local need	n/s
Sarkadi & Rosenqvist (83)	Sharing and learning	Experience-based learning	Joint understanding	Local access to care	n/s
Trento et al. (76)	Peer support	Principles of adult learning	Joint training	Local access to care	System redesign

The abbreviation n/s indicates that no significant information was extrapolated from the studies on these components.

The five components of integration in the context of DSMES are presented to explain how the theoretical framework links to the key concepts identified in the articles.

### Integration Is Constructed Through Interpersonal Relationship

The literature emphasised the importance of interpersonal relationships in DSMES as a key feature to mediate the extent to which people with diabetes participate in self-management behaviours (70, 71, 74, 76, 77, 79, 81–86). It was evident that the different roles and positions as well as the manner in which healthcare professionals developed relationships with people with diabetes influenced these interactions. Intent to support people with diabetes in their self-management behaviour was prevalent in the interactions described in the studies. In one study however, primarily the number of medical appointments was increasing, while participation in programmes of self-management education remained low (71). In that context, the general practitioner-led intervention encouraging people with diabetes to participate in self-management, the participants of the intervention group were three times more likely to achieve their treatment goals within a 12-month period; however, their use of health care services was three times higher than in the control group, while overall participation in DSMES programmes remained low and their self-efficacy did not improve in either the intervention or control groups (71). This contradiction may be due to the way in which some healthcare professionals tend to communicate their support: imparting knowledge as they learned it during their own training, tending to give information and being more verbally active than the chronically ill person with the support needs (74). Furthermore, DSMES programmes with short durations may not be sufficient to increase confidence and self-efficacy in people with diabetes to participate in consultations with healthcare professionals (77).

The extent to which a person-centred approach is put into place by healthcare professionals may relate to their understanding and appreciation of patient participation and thus influence their roles and positioning during their interactions. The involvement of a peer person may bring in new aspects and help the healthcare professionals to understand the patient perspective and train their listening skills (76, 84–86). The different insights of healthcare professionals and peers are complementary and, therefore, might be an ideal training ground for improved DSMES. Furthermore, adding peer support to group consultations helped to maintain favourable clinical and psychological outcomes over a longer time (76). Another option identified in the literature was using reflective strategies in simulations and discussions to encourage healthcare professionals to think about patient disease experiences (72, 81–83, 89). There was some evidence from the literature that experiential participatory learning raised some healthcare professionals’ awareness of their own training needs, although this was not explicitly stated. Observational data obtained from videotaped training sessions, for example, indicated that some healthcare professionals rarely asked open-ended questions to encourage patients’ own problem-solving processes and insufficiently used listening skills which elicited some concern about the implementation of DSMES (82).

All in all, *interpersonal relationship* is critical in the interactions between healthcare professionals and people with diabetes and influences the roles and positions in the DSMES learning experience. This construct is decisive for effectively integrating DSMES into routine care. These relationships may be shaped by whether healthcare professionals have been trained in person-centred care delivery, which may help them support people with diabetes according to their individual needs.

### Integration Is Shaped by the Underpinning Ethos of Programme

An essential factor in shaping how integration was implemented in care delivery related to the prevailing educational ethos and the underpinning psycho-educational theories of DSMES programmes. A myriad of psychological approaches and educational models existed and they were often combined in different ways (70–90). Widely used approaches within these articles included empowerment-based models with problem-solving strategies. Another commonly used theoretical approach combined social-cognitive theory with an emphasis on self-regulation and self-determination. Psychological approaches, for example, the transtheoretical model of behaviour change, some cognitive behavioural therapy techniques and motivational interviewing were combined with adult-based learning techniques, such as experience-based learning and persuasion techniques. It was found that the solid theoretical background of DSMES benefited the person with diabetes because the interactions during consultations were more comprehensible and focused on their needs. At the same time, the interventions of DSMES were clearly structured and thus replicable in population groups with similar needs identified elsewhere by different healthcare professionals.

An important feature was the training of the healthcare professionals who delivered DSMES, although it might have been challenging for some to choose the most suitable strategy from the variety of available strategies; all the more so since the most promising DSMES often involved a combination of different strategies. Furthermore, the literature implied that these forms of person-centred provision may not be sustained over time, especially when provided for a short period and disconnected from the ongoing care experiences of people with diabetes (74, 77). In situations where people with diabetes experience encouragement only in the context of DSMES, they, but also healthcare professionals, may become disengaged because the ethos of self-management support does not infuse into routine care. Rather than pointing at the inadequacy of DSMES, this may indicate a failure to adequately integrate the experience of DSMES with the ongoing patient care.

Altogether, *programme ethos*, which provides the philosophical underpinnings of how education and support are delivered in DSMES, influences how people with diabetes experience the delivery of DSMES and how they integrate self-management behaviour into their daily lives. The ethos of a programme plays an important role in facilitating the translation of programme content into practiced health behaviours. While many DSMES interventions tend to impart knowledge of disease and treatment, learning how to live with the disease may encourage people with diabetes to transfer this experience into their life context because the understanding becomes relevant for them. The extent to which the relevance of DSMES persists beyond the initial initiative may relate to the mechanisms within DSMES that enable people with diabetes to connect with their daily lives, the personal goals they have developed, and with their ongoing interactions with healthcare professionals.

### Integration Is Created Through Shared Learning

Shared learning experiences promoted collaboration between multiple healthcare professionals and helped them to develop a common understanding of DSMES across different settings of care delivery (70, 75, 78, 80, 89). DSMES was provided in a wide variety of settings, including community venues (85), pharmacies (83), primary care (70–73, 77, 81, 82, 84, 86–90), and hospital inpatient and outpatient clinics (74–76, 78–80). From the literature, it appeared that the different settings created specific situations and social relations that consciously or subconsciously resonated when the term DSMES was used and, therefore, influenced how people with diabetes experienced DSMES (74). Some might have perceived hospital settings to represent illness and treatment, while associating community settings with neighbourhood support (74). It is important that healthcare professionals from the various settings exchange information regularly, whether through joint case discussions or coaching (71, 72). Establishing channels of good communication between the healthcare professionals and the different sectors of care is crucial, particularly as the primary care sector is increasingly accountable for diabetes management.

Building up collaborative care requires investments in time and effort. Joining processes and structures for service delivery, open communication and a mutual understanding of DSMES nurtured the collaboration of the different disciplines involved in patient care, which in turn may have improved the patient experience (70–73, 75–78, 80, 82–84, 86, 89, 90). However, multi-professional training for healthcare professionals as a format for conveying an understanding of a person-centred approach to DSMES has not yet been widely used. This is all the more important because DSMES, especially in people with diabetes who have developed late complications, necessitates continuous encouragement so that they learn how to cope with impairments in everyday life.

Overall, the component of *shared learning* refers to the conditions that foster collaborative care and may be promoted in multi-professional trainings of healthcare professionals. This conceptualisation may be extended to include people with diabetes as they relate their experiences to the care setting. Shared learning thus promotes collaboration and an integrated care experience that may be facilitated through coordinated DSMES and delivery support.

### Integration Is Developed Through Adapting to Context

An important feature of DSMES in the literature was how the content and delivery were adapted to local conditions and individual needs (70–74, 76–80, 83, 85, 90). In some situations, linguistic and cultural needs were taken into account to adapt DSMES to the socio-cultural backgrounds of the people with diabetes (75, 85, 88). To this end, professional interpreters or bilingual healthcare professionals adapted the content of DSMES and the information conveyed about disease conditions to the context of specific population groups. In addition, more advice on how to access medical services and community resources related to diabetes was provided to address specific needs.

Considering the values and beliefs of people with diabetes was crucial to help them understand their disease (74). Furthermore, different strategies and modes of delivery addressed the individual preferences of people with diabetes. For example, individualised follow-up was integrated with web-based DSMES because this approach was flexible and could fit the time availability of people with diabetes regardless of where they were located (87, 88). Structured DSMES was also delivered individually or in group settings (76, 80, 83, 90). In some situations, delivery was adapted to the needs of primary care by offering specialist support to improve local access to DSMES (71, 72, 89).

Altogether, *adapting to context* considers the values, beliefs and preferences of people with diabetes and healthcare professionals to enhance their acceptance of DSMES. Reflecting on these conditions is important for the integration of DSMES into the routine care of local health care systems.

### Integration Is Mediated by the Organisation of the Care System

The literature exposed the extent to which DSMES was embedded in the broader health care system (70–73, 75, 76, 80, 87–90). The level of integration was associated with normative mechanisms, such as structured treatment plans with common protocols, shared guidelines and care pathways, and with contextual mediators that represent how DSMES was integrated into the processes and structures of routine patient care (70–73, 75–77, 80, 89, 90).

It was evident that using information technology (IT) to transfer information improved communication between healthcare professionals and people with diabetes (87, 88). Nevertheless, concern was raised about computer access and the necessary e-health literacy that could further disadvantage some people with diabetes. The literature indicated that the integration of technology-enabled tools into routine care required access to and acceptance of the technology, as well as the skills needed to use it, and the necessary precautions to protect data.

Including regular follow-up and recall systems within treatment protocols and guidelines were beneficial for the integration of DSMES into the care delivery processes (70–73, 75, 76, 80, 89, 90). However, in some situations the necessary organisational changes were not implemented, so DSMES was not integrated into routine care and therefore could not contribute their potential to improve patient outcomes even though the healthcare professionals received financial incentives for the provision of DSMES (73).

Overall, DSMES is conveyed by the *care system organisation* and their strategies for integrating DSMES into routine care. Structured treatment plans with defined guidelines, common protocols and care pathways govern the processes of DSMES delivery, and the necessary information transfer may be eased through the use of technology.

### Interactions and Relationships Between the Components of Integration

**Figure 2:**
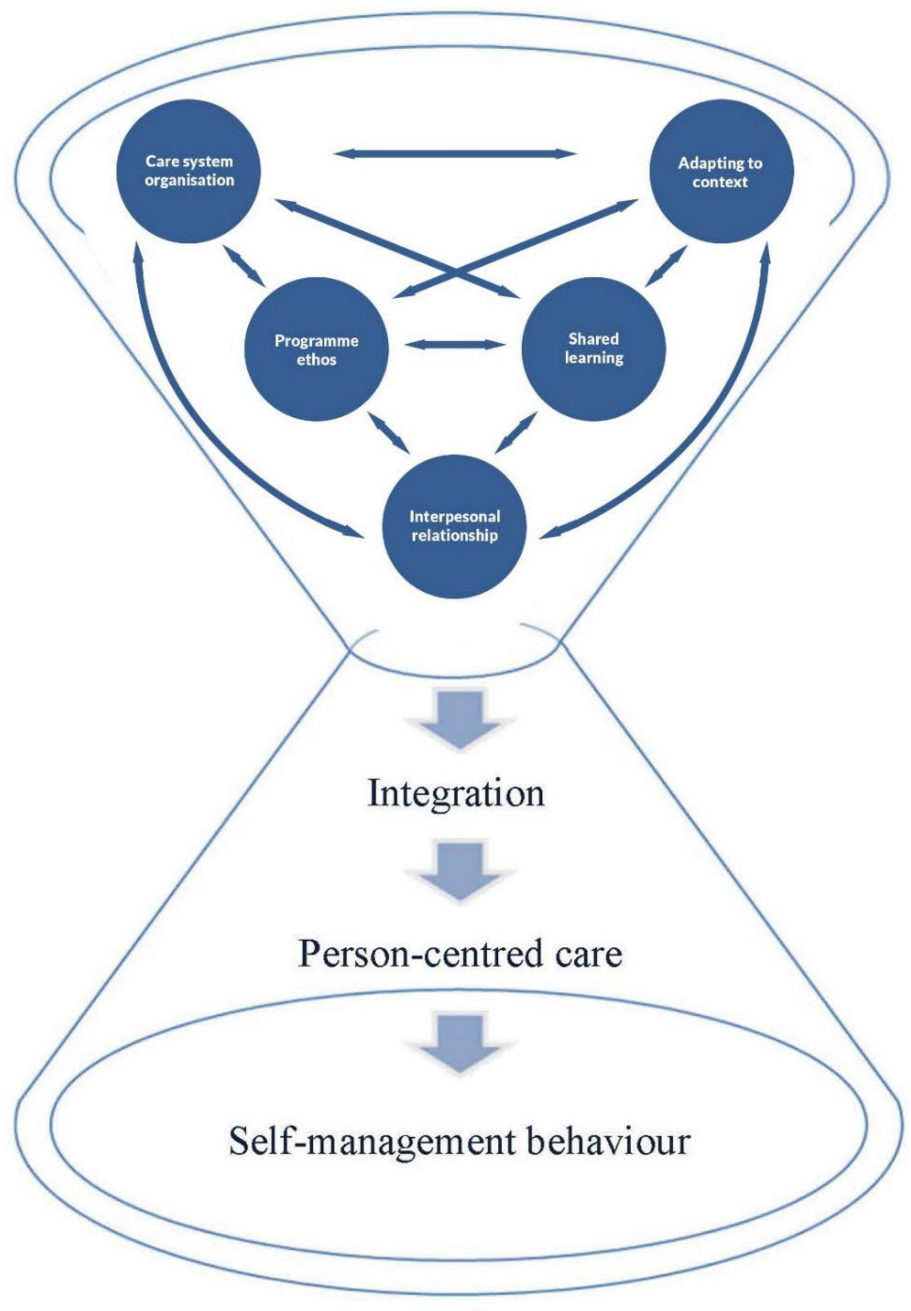
Theoretical model of interacting components influencing the integration of DSMES to affect person-centred care and self-management behaviour in routine care.

The five components of integration were conceived as a cascade-like interaction (20), assuming a systemic non-linear interdependence (see **Figure 2**).

Interactions between the components may influence the delivery of person-centred DSMES and the uptake of self-management behaviour according to the importance and priority given to each individual component. The *interpersonal relationship* component expresses the interactions between the healthcare professionals and the person with diabetes during DSMES and is likely driven by the underlying positions people take in their exchanges, as evidenced by how they participate and contribute. These interactions may interrelate with the other components, for example, by influencing how opportunities for shared learning between healthcare professionals and people with diabetes are encouraged in DSMES. The *shared learning* component, in response, may affect the delivery and implementation of DSMES and be influenced by how the programme ethos, underpinning DSMES, supports self-management behaviour in practice. The approach in the *programme ethos* component, in turn, may guide and influence healthcare professionals’ positions in conducting DSMES. The *adapting to context* component considers the specific situations of the person with diabetes and healthcare professionals and may influence the content and delivery mode of DSMES. This contextualisation addresses the different needs and preferences of a diverse population in DSMES. The model considers the collaborative interactions that are observable in DSMES and delivered by multiple healthcare professionals. These collaborative interactions are reflected in the ways different health care services are linked to each other. The *care system organisation* component in the model illustrates that structures and processes, such as protocols, care pathways, guidelines and IT systems, build the context for the delivery of DSMES and its transfer into routine care. The components of integration are expressed in a systemic approach, with dynamic interactions that may extend or diminish the sense of integration experienced by both the people with diabetes and the healthcare professionals. The importance and prioritisation of individual components influence the context and thereby the conditions that enable healthcare professionals to provide person-centred care and support people with diabetes to develop their self-management behaviours.

This conceptual model of integration was presented and discussed with diabetes specialist nurses (DSNs) in a workshop at the annual conference of the Swiss Diabetes Specialist Nursing Organisation in March 2016 in Berne, Switzerland. Forty-nine DSNs participated in the workshop, which was simultaneously translated between French and German: 19 DSNs spoke French (including 2 DSNs from the Italian-speaking part of Switzerland), and 30 DSNs spoke German (including 1 DSN from the Romansch-speaking part of Switzerland). The workshop was simultaneously translated in order to capture different linguistic interpretations and to directly discuss any differences. The workshop participants agreed on the most important features of each component and identified an opportunity for improvement in the components that were currently receiving low priority in the Swiss context. The priorities given and the consensus reached for components are shown in **Table 2**. It was interesting to note that French-speaking DSNs focused rather on the component of interpersonal relationship while some German-speaking DSNs prioritised the component of the programme ethos underpinning DSMES and suggested arranging the other components around the ethos of DSMES. Overall, the participants agreed that the conceptualisation of the components and their content was fit for purpose and specific for DSMES (67).

**Table 2:** Level of importance given by the participants to components, consensus reached between DSNs from different linguistic areas, and identified opportunities for improvement.

Component	German-speaking DSNs	French-speaking DSNs	Consensus
**Interpersonal relationship**	++++	+++++	Respect and trust are prerequisites for equal relationships
**Programme ethos**	+++++	++++	Biopsychosocial and educational needs are addressed based on priority
**Shared learning**	+	+	Interprofessional training to improve collaboration/involvement is needed
**Adapting to context**	++	+++	Cultural and linguistic specificities are partly considered for easy access
**Care system organisation**	+++	++	System adaptation to promote self-management is needed

DSNs, diabetes specialist nurses; priority given from lowest (+) to highest (+++++); a lower priority represents more opportunities for improvement.

## Discussion

A critical interpretative synthesis was conducted to conceptualise the integration of DSMES into routine care. Five interacting components of integration were identified and described. This conceptualisation contributes to the understanding of integration – which is needed because of the many different interpretations – in the context of DSMES. Prior to the conduct of this review, a definition of the meaning of “integration of DSMES” was missing in the literature. The components broadly cluster around relational, ethical, learning, contextual adapting, and systemic organisational aspects of DSMES. In addition to developing the model, several new findings emerged from the review.

Interpersonal relationships between healthcare professionals and people with diabetes are crucial for their experience of DSMES, this is already widely acknowledged (17, 21, 91–95). However, it might be that healthcare professionals are less aware of how different agendas and expectations shape their interactions with people with diabetes, and in what ways this may create discrepancies in their experiences. Specific training for healthcare professionals could help them to build relationship and provide support that will be beneficial for the person with diabetes. Although person-centred DSMES has already expanded into many care delivery settings, a more patronising approach to patient care, where healthcare professionals adopt a rather authoritarian attitude, is still present and may impede the implementation of person-centred initiatives (96, 97). Integrating the training element of person-centred care delivery for healthcare professionals into any DSMES would therefore improve the conditions for relationship-building, and thus, the participant experience in DSMES.

Structured DSMES use diverse disease models and different approaches related to psycho-educational, learning and behaviour change theories. These aspects are well known in the literature and showed better results the more frequently and intensively they were used and the more the healthcare professionals were trained in implementing DSMES (12, 18). In spite of that, it remains still unclear to what extent the healthcare professionals’ training, especially in therapeutic patient education, is effectively implemented in routine care in terms of building a person-centred relationship. Therapeutic patient education has been evolving in Europe for over 25 years, albeit in different ways, leading in some places to lasting changes in the treatment, education and support of people with diabetes as well as in the training of healthcare professionals and their perceptions of their roles (98–101). For example, in Switzerland, therapeutic patient education has developed strongly in French-speaking Switzerland around the Division of Therapeutic Education for Chronic Diseases of the Geneva University Hospitals (HUG), where specialised postgraduate training courses in therapeutic patient education have been offered to healthcare professionals from different disciplines since 1998 (102). Training healthcare professionals in therapeutic patient education has shown promising results in terms of how DSMES is implemented in clinical practice (103, 104). The helpful implementation of the underlying ethos of DSMES and the associated experiences of care also play an important role in integrating DSMES into the daily lives of people with diabetes. The extent to which self-management behaviours persist beyond one-off patient support is mediated by the interacting components, potentially triggered by mechanisms within DSMES, that enable people with diabetes to reconcile the personal goals they have developed with the demands of their daily lives and the ongoing interactions with healthcare professionals.

The vast diversity of people participating in DSMES creates its own context with specific situations during their exchanges. The features of these exchanges are shaped by the healthcare professionals with their professional experience, knowledge of the disease and priorities in the patient treatments, and by the people with diabetes with their life experience, understanding of the disease and priorities in life. Such interactions may produce disconnects and uncertainties that require negotiation to reach an agreement because they often originate in different priorities (105). Multiple healthcare professionals provide DSMES. Therefore, the context of DSMES also refers to the conditions that encourage collaborative care, promoted in multi-professional trainings of healthcare professionals, where dynamic interactions form the behaviour of a healthcare professional team (106, 107). In addition to the multi-professional training, the inclusion of the person with diabetes in the delivery of DSMES helps the healthcare professionals to better understand what it means to live with diabetes. Interactions with people with diabetes are also an important source of learning for healthcare professionals because interactions based on a narrow biomedical understanding of patient needs may lead to a transfer of knowledge that is not relevant for the person with diabetes, and thus, creating a disconnect for them (108). The conditions for DSMES integration could be further promoted through regular interprofessional training, more opportunities for shared learning also involving people with diabetes, and the provision of support for the interprofessional DSMES delivery, which are not yet sufficiently implemented in many health care services.

The extent to which DSMES adapts the content and delivery mode to the cultural, ethnic, geographic, cognitive and literacy aspects of a population is an important factor for the uptake of DSMES, which is widely acknowledged in the literature (18, 109). Tailoring to a specific population’s needs is even more important when addressing difficult-to-reach populations and those with a low level of health literacy, as they often have complex health needs and are more affected by diabetes complications; integration in such a context may imply, for example, that some people with diabetes need to learn how to navigate the health care system in order to access care (110–112). The way in which healthcare professionals deliver care may also be influenced by their values, beliefs and preferences; thus, their likelihood that they adopt the person-centred approach to DSMES and adapt their care delivery. Such contextual adaptation takes into account the specific situations and experiences of both people with diabetes and healthcare professionals in order to choose the most suitable ways to deliver DSMES. Reflecting on these conditions in the development and improvement processes of DSMES is worthwhile when considering the integration of DSMES into the routine care structures of the local health care system.

The integration of DSMES also depends on the structures and processes of the care system organisation that are available to implement DSMES. In many instances of DSMES implementation, ongoing support and quality assurance are scarce and could benefit from being embedded in structured disease management programmes. It is also important to monitor participation rates to identify groups that may be less likely to attend DSMES, so that adaptations can be made such as directing programmes towards these groups and, where appropriate, changing how they are delivered. There is an ongoing policy shift towards more person-centred DSMES, and it is expected that the strategies used will be most beneficial when integrated into the interdisciplinary structures and coordinated processes of routine care, as advocated, for example, in the chronic care model (113–115). Furthermore, integrated care systems are thought to improve patients’ experiences, their health outcomes, and the use of resources; but so far, the evidence has shown equivocal results, especially with regard to person-centred care delivery (30, 116, 117). The reasons for these inconclusive results are multifactorial and may relate to the imprecise definition of integrated care with its wide range of activities and concepts, but also to the insufficient impact of the collective activities of healthcare professionals in changing the patient health status in their context, as shown, for example, by the inconsistency in patient experiences and outcomes (30).

Integrated care is an organisational form for health care delivery and contains a set of care initiatives aimed at implementing person-centred care to help people manage their chronic conditions (118). DSMES is a care initiative whose integration into routine care is influenced by the identified interacting components and shaped by the priorities of people with diabetes, the healthcare professionals involved and the prevalent conditions within health care systems. The defined components contribute to the development of strategies to improve the patient experiences and outcomes of DSMES. Another important strategy for greater integration of DSMES into routine care is adequate reimbursement structures, especially in care systems without universal healthcare coverage (119).

An organisational context and structures that take into account the patient experiences and outcomes of DSMES also provide improvement opportunities for healthcare professionals. Such approaches enable healthcare professionals to realistically adapt to specific situations and thereby also improve their experience of DSMES. These conditions may take into account the dynamically emerging relationships between the healthcare professionals and people with diabetes during DSMES; and thus, may sustain the person-centred approach promoted by the ethos of DSMES programmes more strongly and for a longer time in routine care. It is expected that contexts, integrating these five components of DSMES into their care provision, will encourage the delivery of person-centred care and the adoption of self-management behaviour, which may lead to improved uptake and impact of DSMES.

### Strengths and Limitations

Using a critical interpretive synthesis approach, we conceptualised and defined five interacting components of integration related to DSMES in routine care. This approach offered the prospect to reframe and reinterpret existing literature through the argument of integration that generated new insights. As in many interpretive syntheses, some of the articles only indirectly addressed the review questions; the synthesis extrapolated data on the integration of DSMES from those articles.

Inherent to critical interpretive syntheses, articles were included based on their conceptual quality, which means that the identified articles provided content relevant to the review questions. We searched multiple databases and used inclusive search terms. The study designs and how the studies contributed to the conceptualisation were included in the review. All articles were discussed and compared in detail in terms of their theoretical contribution to the conceptualisation. The review team supported reflexivity throughout the review process, documented the decision processes and guarded against framing the analysis according to a single perspective. Given the wide range of topics covered by this critical interpretive synthesis, we may have missed relevant studies. Because our aim was conceptual saturation, we consider this interpretive approach acceptable. Furthermore, the components and the model were discussed for their face validity during an interactive workshop at an annual professional meeting with diabetes specialist nurses familiar with DSMES. Though, for further evaluation, study designs with higher validity levels and multi-professional teams will be used. A further limitation of the study inheres to the translation processes in the workshop discussions and their interpretation for this study. While it is a strength of the study that the components and the model were discussed with diabetes specialist nurses from different linguistic and cultural backgrounds as well as from different work settings with the help of professional translators, the workshop’s findings were translated into English. And therefore, the interpretation of the components might be different in a predominantly English-speaking context. The translation processes may alter the interpretation and though not reflect the true understanding.

## Conclusion

Based on this critical interpretative synthesis, the integration of DSMES into routine care is defined as five interacting components related to relational, ethical, learning, contextual adapting, and systemic organisational aspects that interact within and among themselves and manifest in non-linear interactions in the context in which they are presented. However, it remains unclear which mechanisms trigger these interactions; this is the subject of a follow-up study that will be reported elsewhere. Furthermore, more research is needed to evaluate how the professional training of healthcare professionals in person-centred therapeutic patient education affects the components of integration.

## Data Availability Statement

The original contributions presented in the study are included in the article/**Supplementary Material**. Further inquiries can be directed to the corresponding author.

## Author Contribution

CH and AF conceived the study and drafted the key areas for review. CH searched the literature; the results were discussed with CM and AF. CH led the development of the theoretical model in conjunction with CM and AF with expansion from DC. All named authors participated in the preparation of the manuscript, providing written comments on drafts and approving the final version.

## Funding

This study presents independent research funded by an unconditional grant from the Nursing Science Foundation Switzerland.

## Conflict of Interest

The authors declare that the research was conducted in the absence of any commercial or financial relationships that could be construed as a potential conflict of interest.

## Publisher’s Note

All claims expressed in this article are solely those of the authors and do not necessarily represent those of their affiliated organizations, or those of the publisher, the editors and the reviewers. Any product that may be evaluated in this article, or claim that may be made by its manufacturer, is not guaranteed or endorsed by the publisher.
