# Supplementary material for: Integrating Self-Management Education and Support in Routine Care of People With Type 2 Diabetes Mellitus: A Conceptional Model Based on Critical Interpretive Synthesis and A Consensus-Building Participatory Consultation
Source: Front Clin Diabetes Healthc. 2022 Jun 3;3:845547. doi: 10.3389/fcdhc.2022.845547 (PMC10012123; doi:10.3389/fcdhc.2022.845547)
Supplement: Supplementary file 1 [file Table_1.pdf]

Supplementary File 1: Summary of quantitative studies included in the review

Author, year, country	Study design	Purpose/aim	Main professionals and theoretical framework	Intervention	Participants and location	Key concepts/themes	Contributions
Adolfsson *et al.* (2007), Sweden	Randomised controlled trial	Evaluate the impact of empowerment group education in primary care	GP and diabetes specialist nurse; based on empowerment	Five 2.5-hour group sessions with one follow-up session using reflection and feedback among participants<br>Joint HCPs training (5 days in total): simulation; workshop understanding empowerment, motivation and learning principles; practising in four supervised pilot group sessions; and follow-up-meetings	PwT2D in seven primary care centres in Central Sweden (n = 101)	• Reflective peer discussion<br>• HCPs understanding of theoretical concepts and delivery mode	• Peer support<br>• Empowerment<br>• Joint understanding
Carey *et al.* (2014), UK	Non-randomised controlled trial	Evaluate the delivery of the DESMOND diabetes self-management education programme by paired professional and lay educators	Lay educator and HCP; based on self-regulation theory, dual process theory, self-determination theory and social-cognitive theory	Six-hour structured group self-management education programme for PwT2D, delivered either over 1 day or 2 half days<br>Lay educator training (6 days): preparation day; standard DESMOND educator training, joint practice days and training of lay and HCP educator; additional day for lay educator; site visits and quality assessment	PwT2D in four primary care organisations across England and Scotland (n = 260)	• Equal roles of HCPs and peer educators in interactions<br>• Joint training of HCPs and laypeople in programme delivery	• Roles and positions<br>• Combined strategies<br>• Joint training
Deakin *et al.* (2006), UK	Randomised clinical trial	Evaluate a group-based, peer-led self-management programme (X-PERT) in community venues	HCP, peer educator and an (Urdu-speaking) cultural translator in some sessions; based on empowerment (problem-solving strategies)	Six 2-hour weekly group sessions separately conducted for Urdu-speaking South Asian participants with the support of a translator.<br>Joint training: not reported<br>Programme contains written curriculum, visual aids, a "train the trainers" course, evaluation scheme and quality assurance programme	PwT2D with Caucasian and South Asian backgrounds in primary care and community venues in North West England (n = 314)	• Interactions with peers<br>• Peer support by trained layperson<br>• Local access (community)<br>• Linguistic and cultural programme adaptation	• Peer support<br>• Empowerment<br>• Joint training<br>• Community access
Debussche *et al.* (2012), France	Randomised controlled trial	Evaluate quarterly outpatient counselling visits by nurses and dieticians following in-hospital educational sessions	Diabetes specialist nurse and dietician; based on problem-solving strategies	Quarterly lifestyle support in outpatient clinic from Creole-speaking nurses and dieticians after intensive in-hospital group education<br>Initial educational cycle: interactive lectures and focus group discussions (1–2 hours) on six diabetes-related topics as well as physical activity and cooking workshops by a multidisciplinary healthcare team<br>Postal and telephone reminder	PwT2D recruited from two endocrinology departments of the Regional Hospital of Reunion Island (n = 398)	• Linguistic and cultural programme adaptation<br>• Collaboration of multidisciplinary diabetes care team<br>• Regular follow-up (by multidisciplinary team)<br>• Recall system	• Problem-solving<br>• Collaboration in multi-professional team<br>• Linguistic/cultural variations<br>• Recall system

Author, year, country	Study design	Purpose/aim	Main professionals and theoretical framework	Intervention	Participants and location	Key concepts/themes	Contributions
Du Pon *et al.* (2020), the Netherlands	Randomised controlled trial	Evaluate a group-based proactive inter-disciplinary self-management education programme (PRISMA) in primary care	GP, primary care nurse, dietician; based on patient empowerment using self-regulation theory, dual process theory, self-determination theory and social-cognitive theory	Two 3.5-hour group sessions with follow-up in action planning and goal setting in individual consultation with HCPs<br>Joint HCPs training and prior participation in PRISMA programme, communication skills training (motivational interviewing)	PwT2D recruited from eight primary care centres in the Eastern part of the Netherlands (n=203)	• Collaboration of multidisciplinary diabetes care team<br>• Communication skills training<br>• Local access	• Person-centred communication<br>• Empowerment<br>• Joint training<br>• Local access to care<br>• Regular follow-up
Glasgow *et al.* (2012b), USA	Randomised clinical trial	Evaluate internet-based self-management programme with or without additional live support	GP, diabetes educator, dietician, trained bilingual staff; based on social-cognitive theory and social-ecological model	Web access to an interactive self-management programme (in Spanish and English) providing goal setting with automated feedback and prompts as well as information on community resources and a moderated forum<br>Additional support: social support with two personal follow-up calls and three 2-hour group sessions on healthy eating and problem-solving skills, improving communication, and using community resources	PwT2D with multi-ethnic backgrounds in five primary care clinics of managed care organisation in Colorado, USA<br>(n = 463)	• Linguistic and cultural programme adaptation<br>• Virtual programme combined with in-person follow-up<br>• Information about local resources<br>• Service redesign (IT integration)	• Combines strategies<br>• Virtual and individualised support<br>• Information on local options<br>• Service redesign
Goderis *et al.* (2010), Belgium	Randomised controlled trial	Evaluate quality improvement programme to support general practice in chronic care delivery	GP, diabetologist, diabetes specialist nurse, dietician, health psychologist; based on chronic care model and transtheoretical model of change	GP: treatment protocol supported by two postgraduate training sessions, case coaching, annual benchmarking, referral to interdisciplinary team for diabetes education and incentives (€60/patient)<br>Additional training: three monthly follow-ups, stimulated shared care with an interdisciplinary team, two sessions on person-centred communication, motivational interviewing to facilitate behaviour change and peer discussion<br>Patient: free patient education in primary care location or in patient's home by interdisciplinary team; material for measuring blood glucose	PwT2D in 74 primary care practices in Belgium (n = 2256)	• Treatment protocol and shared-care guidelines<br>• Training of HCPs (case coaching)<br>• Learning strategy (person-centred communication)<br>• Reorganisation of local care team<br>• Feedback on performance<br>• Financial incentives	• Person-centred communication<br>• Combined strategies<br>• Interdisciplinary training<br>• Feedback on performance<br>• Treatment guideline
Katon *et al.* (2010), USA	Randomised controlled trial	Evaluate coordinated care management of poorly controlled PwT2D with multiple coexisting conditions	GP, primary care nurse, specialists (psychologist, psychiatrist); based on problem-solving strategies	Guideline-based treat-to-target disease management of multiple coexisting conditions with primary care nurse to support self-care; proactive structured follow-up in primary care every 2 to 3 weeks; educational self-care material<br>Weekly case review and supervision of primary care nurse with a psychiatrist, psychologist and GP; tailored treatment with medication<br>Maintenance plan that includes stress reduction, behavioural goals, use of medications and early symptoms of worsening conditions; monthly follow-up phone calls	PwT2D with multiple conditions (diabetes and/or coronary heart disease and depression) from 14 primary care clinics in an integrated healthcare system in Washington State, USA<br>(n = 214)	• Coordination of multiple care needs<br>• Treatment protocol<br>• Collaboration of GP, primary care nurse and specialists<br>• Learning strategy (person-centred communication)<br>• Regular follow-up	• Person-centred communication<br>• Problem-solving<br>• Collaboration in multi-professional team<br>• Coordination of care needs<br>• Treatment guideline

Author, year, country	Study design	Purpose/aim	Main professionals and theoretical framework	Intervention	Participants and location	Key concepts/themes	Contributions
Ko *et al.* (2007), South Korea	Randomised controlled trial	Evaluate the effectiveness of intensive structured group education with regular follow-up	Diabetologist, diabetes specialist nurse, dietician, ophthalmologist, pharmacist, psychologist, GP and medical rehabilitation specialists; based on cognitive behavioural theory	Five 6-hour diabetes group education sessions during hospital stay addressing disease understanding, treatment skills and behavioural strategies to encourage self-management activities; invitation of family members to attend<br>Regular 3-month follow-up in outpatient clinic by diabetes specialist nurse and physician; reminder on non-attendance<br>Annual 3-hour group session to review self-management	PwT2D recruited in university-affiliated diabetes centre in South Korea<br>(n = 547)	• Collaboration of multidisciplinary diabetes care team<br>• Social support (family, other carer)<br>• Adaptation to local needs<br>• Regular follow-up<br>• Recall system	• Social support<br>• Cognitive behavioural therapy<br>• Collaboration in multi-professional team<br>• Local need<br>• Recall system
Piatt *et al.* (2006), USA	Randomised controlled trial	Evaluate the effect of a multifactorial intervention using the chronic care model	GP, nurse practitioner, physician assistant, psychologist, certified diabetes educator (CDE: nurse, dietician); based on empowerment	PwT2D: Six weekly sessions of structured curriculum-based diabetes self-management training facilitated by CDE, followed by monthly support groups up to 12 months<br>GP: One problem-based learning session, case coaching; CDE for consultation in primary care on "diabetes day"<br>Chart reviews against national standard	PwT2D from 11 primary care practices in an underserved suburb of Pittsburgh, Pennsylvania, USA<br>(n = 119)	• Training of HCPs (problem-based learning, case coaching)<br>• Adaptation to local needs<br>• System redesign<br>• Treatment protocol<br>• Feedback on performance	• Empowerment<br>• Joint training<br>• Local need<br>• Treatment guideline
Russell *et al.* (2013), Australia	Non-randomised controlled trial	Evaluate an integrated primary/specialist community care model for complex T2D management	Diabetologist, GP with advanced skills in diabetes care, diabetes specialist nurse, dietician, psychologist, podiatrist; based on empowerment	Weekly multidisciplinary clinic with co-consultation of diabetologists and GP with advanced skills and PwT2D in primary care<br>Postgraduate online training of GP in advanced diabetes care, with case coaching of GP and primary care nurse<br>Biweekly telephone contact to intensify insulin treatment, follow-up phone calls at 6-week and 3-month intervals by diabetes specialist nurse	PwT2D from a tertiary hospital in lower socio-economic suburb of South Brisbane, Australia<br>(n = 330)	• Training of HCPs (postgraduate online training, case coaching)<br>• Relationship of HCPs and patients in multidisciplinary care<br>• Local access to diabetes care (co-consultation)<br>• System redesign<br>• Regular follow-up	• Relationships in multi-professional care<br>• Empowerment<br>• Joint training<br>• Local access to care<br>• Service redesign
Rygg *et al.* (2012), Norway	Randomised controlled trial	Evaluate a locally developed ongoing group-based diabetes self-management education programme for PwT2D	Diabetes specialist nurse, physician, physiotherapist, dietician, PwT2D; based on health education using problem-solving strategies and brief solution-focused therapy	Three 5-hour weekly sessions in outpatient clinics led by diabetes specialist nurses with input from multiple HCPs and experienced PwT2D; extended break time for social interactions<br>Introductory lecture followed by interactive learning and skills training on disease, physical activity, glycaemic control and problem-solving; group discussion of patient experience	PwT2D from two hospital settings within the same hospital trust in Central Norway<br>(n = 146)	• Locally developed programme<br>• Patient referral from GPs<br>• Collaboration of diabetes team and PwT2D	• Combined strategies<br>• Joint training<br>• Local need

Author, year, country	Study design	Purpose/aim	Main professionals and theoretical framework	Intervention	Participants and location	Key concepts/themes	Contributions
Sarkadi & Rosenqvist (2004), Sweden	Randomised controlled trial	Evaluate the effectiveness of an experience-based group educational programme	Pharmacist, diabetes specialist nurse; based on experience-based learning	PwT2D: monthly group meetings to discuss group questions based on experience and individual experiments; sharing diaries and reflecting on practical aspects (preparing food, self-monitoring, walking, choice, emotional aspects) within the group<br>HCP training (3 days): same experience-based learning using identical educational materials (video, booklet, game) as used in the programme for PwT2D<br>Continuous HCP training with regular follow-up meetings every 6 months	PwT2D from participating pharmacies in Sweden (n = 77)	• Learning strategy (sharing of views, beliefs and attitudes)<br>• Training of HCPs (reflection on experience-based learning)<br>• Local access to diabetes care	• Sharing and learning<br>• Experience-based learning<br>• Joint understanding<br>• Local access to care
Trento *et al.* (2010), Italy	Randomised controlled trial	Evaluate the transferability of group self-management education	Physician, nurse, dietitian, educator in the role as facilitator; based on principles of adult education	Seven 1-hour group sessions every 3–4 months on diabetes-related topics; programme may be repeated for reinforcement after 2 years<br>PwT2D training with group work, hands-on activities, problem-solving, real-life simulations and role playing in same group to support group cohesion and interpersonal relationships<br>HCP training in using theoretical framework, teaching manual and materials	PwT2D from 13 hospital-based diabetes outpatient clinics in Italy (n = 815)	• Training of HCPs (understanding theoretical framework, pedagogical principles)<br>• Peer support (identifying with group, disease and activity)<br>• Group care in local service delivery<br>• Service redesign<br>• Regular follow-up	• Peer support<br>• Principles of adult learning<br>• Joint training<br>• Local access to care<br>• System redesign

GP: general practitioner; HCPs: healthcare professionals; PwT2D: people with type 2 diabetes
