# Supplementary material for: Integrating Self-Management Education and Support in Routine Care of People With Type 2 Diabetes Mellitus: A Conceptional Model Based on Critical Interpretive Synthesis and A Consensus-Building Participatory Consultation
Source: Front Clin Diabetes Healthc. 2022 Jun 3;3:845547. doi: 10.3389/fcdhc.2022.845547 (PMC10012123; doi:10.3389/fcdhc.2022.845547)
Supplement: Supplementary file 2 [file Table_2.pdf]

Supplementary File 2: Summary of qualitative studies included in the review

Author, year, country	Study design	Purpose/aim	Type of data collection	Themes in articles	Participants	Key concepts/themes	Contributions
Adolfsson *et al.* (2008), Sweden	Qualitative content analysis	Explore patients' experiences of participating in an empowerment group education programme or receiving individual counselling	Semi-structured individual interviews	Relationships:<br>• Unequal participation in interactions using an instructive approach<br>• Equal contribution in interactions using an empowerment strategy<br>Learning:<br>• Following advice and recommendations in instructions<br>• Participating in learning process in empowerment<br>Controlling the disease:<br>• Externally controlled by professional expertise observed in instructions<br>• Internally controlled by understanding how to actively participate in self-management observed in empowerment	PwT2D previously participating in RCT (Adolfsson *et al.* 2007) (n = 28)	• Positions of HCPs and patients in interactions<br>• Learning style in programme delivery<br>• Understanding of self-management	• Roles and positions<br>• Empowerment<br>• Joint understanding<br>• Varieties of learning
Balcou-Debussche and Debussche (2009), France	Qualitative content analysis using constant comparative approach; ethnographic observation	Assess the effects of self-management education on the individual practices of patients	Semi-structured individual interviews; ethnographic observations of interactions in hospital and at participants' homes	The hospital system:<br>• In the hospital, HCPs recommend treatments for PwT2D to follow<br>• Subordinate role of PwT2D to medical power<br>The suspension of reality in hospital:<br>• HCPs manage disease and PwT2D cooperate: "I was very well, as though the diabetes had disappeared"<br>From hospital to home:<br>• Implementation of learning into social, economic and family environment: "If you don't eat any cake, they ask if there is something wrong with it"	PwT2D participated in same delivery location as in RCT (Debussche *et al.* 2012)<br>(n = 42)	• Delivery location of self-management education<br>• Responsibility for disease management<br>• Transfer of learning into routine	• Disease responsibility<br>• Problem-solving<br>• Transfer of learning<br>• Different locations
Glasgow *et al.* (2012a), USA	Mixed methods using thematic content analyses	Evaluate the extent to which a web-based self-management programme was integrated into patients' primary care	Patient satisfaction survey with four open-ended questions; qualitative interviews with physicians; chart reviews of participants; documentation of process implementation	Themes in GP interviews:<br>• Reinforce behavioural goals<br>• Easy access to information<br>• Integrate with existing programmes<br>• Concerns related to computer access and literacy	• PwT2D participa-ting in intervention groups in RCT (Glasgow *et al.* 2012b) (n = 331)<br>• GPs in five primary care clinics of integrated managed care organisation in Colorado, USA (n = 11)	• Reinforcement strategy through in-person follow-up<br>• Service redesign (IT integration)<br>• Acceptance and/or compatibility of IT resource	• Combines strategies<br>• Individualised support strategy<br>• IT resources<br>• Service redesign

Author, year, country	Study design	Purpose/aim	Type of data collection	Themes in articles	Participants	Key concepts/themes	Contributions
Goderis *et al.* (2009), Belgium	Thematic analysis with theory-based deductive coding using an imple-mentation model	Evaluate barriers and facilitators to high-quality diabetes care as experienced by primary care physicians	Semi-structured individual interviews	Barriers to high-quality diabetes care:<br>• Lack of knowledge and awareness of GPs practice performance<br>• Scepticism about evidence-based treatment and shared-care collaboration<br>Factors facilitating change:<br>• Treatment protocol, postgraduate education, case coaching, benchmarking feedback<br>• Increased contact and communication with colleagues from other disciplines<br>• Role redesign, reassigning responsibility	GPs participating in RCT (Goderis *et al.* 2010) (n = 20)	• Training of HCPs (interdisciplinary teamwork)<br>• Treatment protocol and shared-care guidelines<br>• Adaptation to local needs<br>• Service redesign	• Combined strategies<br>• Interdisciplinary training<br>• Local need<br>• Service redesign<br>• Financial incentives
Hepworth *et al.* (2013), Australia	Thematic analysis	Explore a new model of integrated primary/secondary care for type 2 diabetes management	Semi-structured individual interviews	Person-centred care:<br>• Accessibility in community<br>• Delivery within a positive healthcare environment<br>Effective multi-professional teamwork:<br>• Clear communication<br>Empowering patient:<br>• Relationship (HCPs and patients)<br>• Patients seeing themselves as part of team-based care	PwT2D previously participating in non-randomised controlled trial (Russell *et al.* 2013) (n = 10)	• Local access to diabetes care<br>• Collaboration with multidisciplinary diabetes care team<br>• Relationship of HCPs and patients in multidisciplinary care<br>• Regular follow-up	• Relationships in multi-professional care<br>• Empowerment<br>• Collaboration in multi-professional team<br>• Local access to care<br>• Regular follow-up
Mandalia *et al.* (2014), UK	Thematic analysis using comparative and framework approaches	Elicit the views of key stakeholders (patients and educator) about using lay people and professional educators with equal roles to deliver diabetes education	Telephone interviews	• Differences in knowledge of HCP and lay educator<br>• Peer status in relation to disease experience<br>• Feasibility of using lay educators in self-management education	PwT2D as well as lay and HCP educators previously participating in non-randomised controlled trial (Carey *et al.* 2014) (n = 27)	• Complementary role functions of HCPs and peer educators<br>• Peer support (identification with common experience)	• Peer support<br>• Combined strategies<br>• Joint understanding
Rygg *et al.* (2010) Norway	Systematic text condensation (thematic cross-case analysis)	Explore reasons for participating in group-based diabetes self-management education for patients with type 2 diabetes	Semi-structured focus groups and individual interviews	Experiencing practical problems:<br>• Insufficient answers from HCPs<br>• Contradictory information from different sources<br>Feeling insecure:<br>• Lacking communication with other PwT2D<br>• Missing confirmation about treatment implementation	PwT2D from self-management education groups, some previously participating in RCT (Rygg *et al.* 2012) (n = 22)	• Local access to peer group<br>• Peer support (practical support regarding problem-solving strategies)<br>• Social support (experiencing confirmation within a group)	• Social support<br>• Combined strategies<br>• Joint understanding<br>• Local access to care

GP: general practitioner; HCP: healthcare professional; PwT2D: people with type 2 diabetes; RCT: randomised controlled trial
